# Patient safety climate profiles across time: Strength and level of safety climate associated with a quality improvement program in Switzerland—A cross-sectional survey study

**DOI:** 10.1371/journal.pone.0181410

**Published:** 2017-07-28

**Authors:** Anna C. Mascherek, David L. B. Schwappach

**Affiliations:** 1 Patient Safety Switzerland, Zurich, Switzerland; 2 Institute of Social and Preventive Medicine (ISPM), University of Bern, Bern, Switzerland; University Medical Center Goettingen, GERMANY

## Abstract

Safety Climate has been acknowledged as an unspecific factor influencing patient safety. However, studies rarely provide in-depth analysis of climate data. As a helpful approach, the concept of “climate strength” has been proposed. In the present study we tested the hypotheses that even if safety climate remains stable on mean-level across time, differences might be evident in strength or shape. The data of two hospitals participating in a large national quality improvement program were analysed for differences in climate profiles at two measurement occasions. We analysed differences on mean-level, differences in percent problematic response, agreement within groups, and frequency histograms in two large hospitals in Switzerland at two measurement occasions (2013 and 2015) applying the Safety Climate Survey. In total, survey responses of 1193 individuals were included in the analyses. Overall, small but significant differences on mean-level of safety climate emerged for some subgroups. Also, although agreement was strong at both time-points within groups, tendencies of divergence or consensus were present in both hospitals. Depending on subgroup and analyses chosen, differences were more or less pronounced. The present study illustrated that taking several measures into account and describing safety climate from different perspectives is necessary in order to fully understand differences and trends within groups and to develop interventions addressing the needs of different groups more precisely.

## Introduction

Safety climate (SC) has become a well-established context variable in the analysis of work environment. Safety culture refers to shared beliefs, values, attitudes and behaviour regarding safety within an organization [1] whereas patient safety climate is defined as „the measurable components of safety culture”[2]. It has been shown in a variety of studies that safety climate plays an important role in reducing or preventing patient safety incidents such as treatment errors or readmissions [3–5]. Medication errors, for example, are less frequent in units with high safety climate [6]. Also, 30-day-hospital readmission rates are lower for patients treated in units where frontline staff rate safety climate as high [5].

### Assessment of safety climate

Although safety climate has been recognized as one important aspect for ameliorating patient safety, studies rarely provide in-depth analysis of climate data. The main body of research focuses on mean level of climate, its association with outcomes measures or mean level differences between groups or time points [7–9]. Individual ratings are almost always aggregated to means. Mean values are then used as indicator for high or low safety climate. While it has been widely acknowledged that safety climate may be perceived differently in different units or may vary between hierarchical and professional groups even within one organization [8,10,11] it is mostly neglected that safety climate may also be perceived differently *within* one unit or group. As a helpful approach, the concept of “climate strength” is beginning to gain attention of patient safety climate researchers [12]. The notion “climate strength” is established and has been studied in organisational research [13,14]. As opposed to “level”, which describes safety climate on mean-level, “strength” refers to the degree of consensus among members within a group. Strong climate indicates strong consensus between the raters concerning their perception of safety climate, whereas weak climate indicates variability between raters. Within-group agreement (climate strength) has been promoted as an additional dimension of analyses. It has been shown that climate strength moderates the relationship between for example organizational climate and customer orientation or organizational climate and absenteeism. To analyse the question of whether a collective shift in a cohesive group towards a more favourable climate or rather the convergence of perceptions within a group even on a lower level is more important, strength and shape need to be taken into account.

### Climate strength and patient safety

In the realm of patient safety research and the reduction of patient safety incidents the concept of climate strength is still new [12,15]. In their work, Ginsburg and Oore strongly recommend the use of”safety profiles” to describe safety climate within a given group, including level, strength and shape as instructive measures describing the climate in a given unit. In their study, Ginsburg and Oore investigated safety climate in 24 emergency departments (ED) in Canada. Their results illustrate that in different ED’s with the same safety climate rating on mean-level, diverse subgroups and different degrees of interrater-agreement come to light when additionally safety climate shape and strength are investigated [12]. Zohar reports that a positive safety climate was associated with safety behaviour concerning medication safety and emergency safety behaviour. Climate strength was of additional explanatory power insofar as strong climate even further predicted safety behaviour concerning medication and emergency situations [16]. Generally, studies investigating climate strength consistently report strong climate as a positive aspect as opposed to weak climate. This is true even for strong negative climate as this represents an unanimous team perception, which as a whole might be open for interventions to improve safety climate. Weak climate, however, reflects disagreement within a group which leads to inconsistent behaviour and is more difficult to address by interventions.

### Safety climate and quality improvement programs

Safety climate is a common parameter evaluated in quality improvement programs. It is expected that such programs will have positive effects on (mean) safety climate ratings. However, it is unknown whether differences on mean level necessarily are the only or the genuine effect of improvement programs on safety climate. In particular as studies often fail to show any changes on mean level even if analyses are conducted on unit-level [17]. It is possible that effects of improvement programs rather affect climate strength and shape. Activities within the scope of a quality improvement program might support the establishment of shared mental models concerning safety within units, hence, leading to stronger climate, i.e., consensus among group members, even if climate on mean level remains unaffected.

Although the analysis of climate profiles is becoming more visible in the literature, to the best of our knowledge, no studies analysing patient safety climate profiles across time points exist. Hence, the purpose of the present study was to add to the growing body of research by analysing climate level and strength in different groups across time. In the present paper, differences in climate profiles across time are analysed in two hospitals participating in a large national quality improvement program. We analysed differences on mean-level, strength and shape between the two large hospitals in Switzerland at two measurement occasions (15 months). Analyses focussed on the investigation of possible climate differences between time-points within subgroups. Although subgroups were not large enough to analyse all theoretical possibilities, pronounced subgroups existed to exemplary illustrate the advantage of profile analyses over single-measure mean-level analyses. Data were collected within the scope of a large national improvement program; hence, differences in climate might indicate effects of the program. In the present study we specifically tested the following two hypotheses. First, are there mean-level differences in the evaluation of safety climate between the two measurement occasions (conventional approach)? And, second, how does the variation of safety-climate-evaluations change within subgroups across time even if no mean-level differences might be observable (profile approach).

## Method

### Design

A national quality improvement program was scheduled between summer 2013 and summer 2015 by the Swiss Patient Safety Foundation [18,19]. The main objective of the program was the implementation of the surgical checklist in Switzerland. Part of the program consisted of a specific improvement intervention with 10 participating hospitals. For the purpose of the present study, two hospitals were chosen out of the ten for several reasons. First, within-hospital samples were large enough to warrant further division into subgroups. Second, hospitals had special characteristics that made them interesting candidates for exploratory analyses. Hospital one was a group of three hospitals merged under one umbrella brand. Thus, the question was whether regional differences were more important than the common umbrella brand. Hospital two was a large university hospital and included staff working either on ward or in operating rooms (OR). Hospitals were contractually bound to implement the checklist and execute mandatory activities during the program such as training of checklist use, education of staff, and local adaptation of checklist. Also, explicit support by leadership, promotion by local champions, and establishing a cross-professional team were mandatory components of the program. Additionally, to facilitate exchange and learning between hospitals, 4 mandatory workshops were held during the 2 years of program execution. The implementation program aimed at: first, comprehensively establish the use of the surgical checklist at every procedure in every patient. Second, on a more general level, it aimed at raising awareness concerning patient safety issues and the opportunities for staff to improve patient safety in every day routines. Effects of the intervention, the training and use of the surgical checklist as well as the general awareness of the importance of patient safety were hypothesized to not only show in the actual use of the checklist but also in the evaluation of safety climate.

Data for the present study were collected with a questionnaire during the program at two time points each. The time between data collections was 15 months. Print versions of the survey were sent to hospitals and locally distributed. The survey was provided in German and French. The survey sample consisted of all members of the OR teams of the participating hospitals (doctors, nurses, scrub nurses, surgical technicians, and attendants for surgical positioning) and ward staff (doctors, nurses, nursing assistants, and further professionals who were subsumed under “others” involved in the pre- and postoperative care of surgical patients). Subjects were invited to participate by the hospitals’ project teams and repeatedly reminded throughout the data-collecting period. As the study was conducted as part of a quality improvement project the study was exempted from review by the cantonal ethics committee (BASEC-Nr. Req-2016-00758). Questionnaires were returned anonymously to the study team. In order to match questionnaires of the same individual returned at the two measurements participants were asked to generate a code themselves and provide this code in the questionnaire. Individuals were instructed to use certain fragments of their parents’ birthdates and initials so that the likelihood of identical codes was minimized while retaining anonymity.

### Safety Climate Survey

The Safety Climate Survey was applied [20,21]. The original version of the Safety Climate Survey was translated by a professional translator from English to German and back-translated to English by a second translator. Differences in translation and back-translation were discussed and resolved by the research team. The survey was also translated to French and proofread by bilingual researchers in French (for the wording of all survey-items see S1 Table). Two versions (“OR” and “ward”) were developed differing in the wording of single items referring to the specific working area. The translated Safety Climate Survey showed good internal consistency: Cronbach’s alpha_Total_ = 0.85 (95% CI: 0.84–0.86); Cronbach’s alpha_German_ = 0.86 (95% CI: 0.85–0.88); Cronbach’s alpha_French_ = 0.84 (CI: 0.82–0.86). Further details on survey development and validation are reported elsewhere [8]. The questionnaire consisted of the 19 items of the Safety Climate Survey and was rated on a 5-point Likert-scale from 1 = “disagree strongly” to 5 = “agree strongly”. At the end of the survey, socio-demographic variables were assessed.

### Data analyses

Negatively worded items were reverse coded to insure that higher scores indicated a more positive assessment of safety climate for every item. All analyses were conducted on scale-level. We calculated three different measures in order to fully describe differences and variations of safety climate, namely, mean-level, interrater agreement within groups (rwg, James at al., [22]), the percentage of problematic response (PPR, Singer at al.[10]), and frequency histograms for the different subgroups of interest. PPR refers to the percentage of individuals that scored low on the respective scale. Answers ≤ 2 on the 5-point Likert scale were treated as ‘problematic’ response. Accordingly, ‘a low PPR is indicative of a high safety climate’ [11]. Furthermore, a PPR higher than 10% is assumed to be inconsistent with an optimal level of safety climate within an organization. Mean-level differences were examined using ANOVA. If homogeneity of variance was violated, F* was additionally estimated to double check and control for inflated Type-I-error rates. F* is a measure similar to oneway ANOVA, however yields robust p-values even under the violation of homogeneity of variance. Scheffé tests were applied to correct for multiple testing. Rwg(j) has been developed by James et al. [23] as a measure for within-group agreement. LeBreton and Senter as well as Klein et al. [24,25] added to the applicability of the measure in applied research by providing cut-off scores and discussing significance tests, different null distributions and other questions that might occur in the course of applying the Rwg measure. Although the normal standard deviation (SD) is also accepted as a measure, LeBreton and Senter argue for the use of Rwg, as SD rather represents disagreement than agreement [24]. In an analysis which focusses on the extent of agreement, the use of Rwg is recommended. Rwg-values between 0.51 and 0.70 indicate moderate interrater-agreement; values above 0.71 indicate strong agreement [24]. Statistical significance criteria were provided by Smith-Crowe et al. [26], and indicate, depending on sample size, number of response scale categories, and chosen null distribution, whether agreement is due to chance or not. For details on the exact calculation of rwg, see James et al. [23]. As an estimate for the expected variance we used the uniform null distribution as this is the most common reference. Frequency histograms additionally illustrate the rwg-value as they display the distribution of the individual ratings on the scale. No subgroups <10 respondents were analysed separately. Analyses were conducted separately for the two time-points. Hospital 1 was additionally divided by location as three small regional hospitals from slightly different locations were merged together under the umbrella brand of hospital 1. No additional subgroups with regard to workplace could be established for hospital 1 due to sample size. Although this would have been desirable, sample size of the different subgroups was not large enough. We chose the division into location-groups over the division of workplace-groups for theoretical reasons. Hospital 2 was a large university hospital with only one location. For hospital 2, subgroups were divided by workplace (OR/ward). Although in the OR different specialties are present, it was more reasonable to analyse the complete sample only divided by ward/OR than to divide by subspecialty and, hence, exclude specialties with less than 10 individuals. For the purpose of the present study, analyses on ward/OR-level were the most reasonable. All analyses were conducted using STATA v13.1 [27].

## Results

### Sample

1209 individuals returned the questionnaire. From this 1209, 16 had to be excluded due to ambiguous responses. Hence, 1193 individuals were included in the analyses. At the first data assessment (T1), 670 individuals (hospital 1: 299; hospital 2: 371) completed the survey. At the second data assessment (T2), 523 individuals (hospital 1: 229; hospital 2: 294) completed the survey. Hence, with regard to sample size, samples are balanced between hospitals at both measurement occasions. Additional sample characteristics separated by time-points are presented in Table 1. As only “profession” differed significantly between time points, samples were considered as being comparable with respect to socio-demographic variables.

**Table 1:** Sample characteristics (*N*_*total*_ = 1193) *N* for subsamples are: *n*_*t1*_ = 670; *n*_*t2*_ = 523. Data not adding up to 100% are due to missing values.

Characteristic†	T1		T2	
	*n*_t1_ = 670	%	*n*_t2_ = 523	%
Survey language				
German	406	60.6	311	59.5
French	264	39.4	212	40.5
Gender				
Male	201	30.0	183	35.0
Female	454	67.8	326	62.3
Mean age in years	38.8 (SD: 10.3)		38.8 (SD: 10.1)	
Profession*				
Physician	194	29.0	188	36.0
Nurse	446	66.6	310	59.3
Managerial function				
Yes	193	28.8	143	27.3
No	454	67.8	359	68.6
Years of professional experience				
0–2	69	10.3	68	13.0
2–5	128	19.1	90	17.2
5–10	131	19.6	109	20.8
10–20	164	24.5	125	23.9
More than 20	159	23.7	119	22.8
Hours of direct patient care per week				
0	99	14.8	77	14.7
0–8	101	15.1	76	14.5
8–16	73	10.9	53	10.1
16–24	81	12.1	75	14.3
24–32	87	13.0	69	13.2
32–40	121	18.1	89	17.0
More than 40	85	12.7	61	11.7

^†^ *Note*: Characteristics marked with “*” differ significantly between time-points on *p* < .05.

### Safety climate

In order to fully describe safety climate on different levels, we analysed mean-level, PPR, and rwg.

#### Mean-level

Differences on mean-level were analysed from different perspectives. First, we analysed whether safety climate on mean-level differed between time-points in each hospital on general level. In hospital 1 no significant differences emerged over time (*M*_*T1*_ = 3.7; *SD*_*T1*_ = 0.6; *M*_*T2*_ = 3.7; *SD*_*T2*_ = 0.6; *F*_(1,465)_ = 0.53, *n*.*s*.). In hospital 2, however, differences reached significance between time-, however, remaining small (*M*_*T1*_ = 3.7; *SD*_*T1*_ = 0.6; *M*_*T2*_ = 3.8; *SD*_*T2*_ = 0.6; *F*_(1,663)_ = 7.92, *p*<0.05). We then further analysed whether differences between subgroups within hospital 1 and 2 emerged. In hospital 1, differences between the three regional locations were present at time-point 1 (*M*_*L1*_ = 3.6; *SD*_*L1*_ = 0.6; *M*_*L2*_ = 4.0; *SD*_*L2*_ = 0.3; *M*_*L3*_ = 3.7; *SD*_*L3*_ = 0.6; *F*_(2,260)_ = 4.71, *p*<0.05). Although within locations, no differences emerged across time-points, differences between locations were not significant at time-point two anymore, indicating convergence between locations (*M*_*L1*_ = 3.6; *SD*_*L1*_ = 0.6; *M*_*L2*_ = 4.0; *SD*_*L2*_ = 0.8; *M*_*L3*_ = 3.6; *SD*_*L3*_ = 0.6; *F*_(2,201)_ = 2.12, *p*<n.s.). Unfortunately, sample size in hospital 1 was too small to additionally analyse safety climate differences between ward and OR staff.

Next, we analysed whether differences in subgroups emerged in hospital 2. As hospital 2 was not divided into different regional locations, we analysed differences between ward and OR as well as differences between professions. Here, again, we first examined within group differences across time-points and, second, examined convergence or divergence between groups at T1 and T2. Looking at differences in the OR, we found a significant increase in safety climate across time-points (*M*_*T1*_ = 3.7; *SD*_*T1*_ = 0.6; *M*_*T2*_ = 4.0; *SD*_*T2*_ = 0.5; *F*_(1,262)_ = 8.04, *p*<0.05). No differences emerged for safety climate on the ward (*M*_*T1*_ = 3.7; *SD*_*T1*_ = 0.6; *M*_*T2*_ = 3.7; *SD*_*T2*_ = 0.6; *F*_(1,399)_ = 1.47, *p*<n.s). We then analysed the difference between safety climate ratings on ward and OR at T1 and T2 and found no significant differences at T1 (*M*_*OR*_ = 3.7; *SD*_*OR*_ = 0.6; *M*_*ward*_ = 3.7; *SD*_*ward*_ = 0.6; *F*_(1,369)_ = 2.46, *p*<n.s.) but significant differences at T2 (*M*_*OR*_ = 4.0; *SD*_*OR*_ = 0.5; *M*_*ward*_ = 3.7; *SD*_*ward*_ = 0.6; *F*_(1,292)_ = 12.53, *p*<0.05), indicating divergence with respect to climate at T2 between ward and OR.

#### PPR

Percent problematic responses were analysed as it is a common measure within the safety climate literature. PPRs higher than 10% are regarded as indicating need for improvement. As can be seen from Table 1, PPRs are well below 10% in all groups across time-points. Means and PPRs are presented in Table 2.

**Table 2:** Means, standard deviations (SD), percent problematic response (PPR), and interrater-agreement (rwg-values) of the safety climate scale for each subgroup at time-point 1 and 2.

	Mean (SD)		PPR*		Rwg**	
Subgroup	T1	T2	T1	T2	T1	T2
Hospital 1	3.7 (0.6)	3.7 (0.6)	1.34	3.06	0.85	0.80
Location						
Location 1	3.6 (0.6)	3.6 (0.6)	2.2	3.9	0.84	0.80
Location 2	4.0 (0.3)	4 (0.8)	0	7.7	0.84	0.82
Location 3	3.7 (0.6)	3.6 (0.6)	0	0	0.88	0.75
Hospital 2	3.7 (0.6)	3.8 (0.6)	2.96	1.36	0.82	0.85
workplace						
Ward	3.7 (0.6)	3.7 (0.6)	3.49	1.74	0.82	0.85
OR	3.7 (0.6)	4.0 (0.5)	2.11	0.82	0.83	0.85

* PPR = percent problematic response. Percentage of individuals answering ≤ 2 on the scale. PPRs higher than 10% indicate problematic safety climate. Percentage is not independent of sample size;

**All Rwg-values differ significantly from chance.

#### Rwg

In a next step, we analysed subgroups with respect to interrater-agreement. Rwg-values for safety climate in each subgroup are presented in Table 2. All rwg-values were, according to Smith-Crowe et al. [26] significant, indicating that interrater-agreement was greater than chance. Also, all rwg-values indicate strong agreement within the units analysed. In hospital 1, however, rwg’s at T2 were weaker than at T1. Although on mean-level, differences became smaller or remained stable, on agreement-level, discrepancies became stronger. In hospital 1, ratings within groups appear to drift further apart. The opposite is true for hospital 2. Here, at T2 rwg’s were generally stronger than at T1, indicating that in addition to small increases in safety climate on mean-level, there were also increases in the extent of interrater-agreement within groups.

#### Frequency histograms

In a last step of the analyses, we generated frequency histograms of the climate ratings in order to get an impression of the shape and the distribution of the ratings across the scale. Frequency histograms are displayed in the supporting information S1 Fig. Generally, frequency distributions in all subgroups are of similar shape. A central tendency is evident, with no clear extreme or subgroups within the defined groups. Hence, although the shape slightly varies across groups and time-points, this analyses lead to the conclusion that within the defined groups no additional subgroups are evident.

## Discussion

In the present study we aimed at investigating whether differences in safety climate emerged between measurement occasions. In addition to the analysis of mean-level differences, we also took PPR, rwg, and shape into account. These measures were included into the analyses as they provide additional information and, hence, allow for a complete description of the construct.

Overall, PPR was well below the critical value of 10% in all subgroups across time. Although PPR is not independent of sample size (the smaller the group the stronger the influence of every individual), results of the present study indicate that safety climate ratings are generally acceptable according to this measure. No explicit indication for a needed intervention to improve safety climate emerged from the PPR-values. However, in the present study, PPR represented only one indicator of the safety climate profile. Taking rwg, mean-level, and shape also into account is a necessary step to get an in-depth impression of differences in safety climate across time-points. Overall, small but significant differences on mean-level emerged for some subgroups. Also, although agreement was strong at both time-points within groups, tendencies of divergence or consensus were present in both hospitals.

In hospital 1, no significant mean-level differences emerged on hospital level. Hence, between time-points safety climate remained stable overall. However, analysing the three regional locations separately, a slightly different picture emerged. At first measurement occasion, locations differed significantly. Although within each location no significant difference emerged between measurement occasions, the difference between locations was not significant anymore at second measurement occasion. This illustrates that differences became smaller and locations moved closer together. One might interpret this result as homogenization of safety climate between locations. On mean-level, results indicate that differences between locations became smaller. Thus, safety climate became more homogeneous within the hospital group. However, when climate strength and shape are considered, a slightly different picture emerges. All calculated rwg-values for hospital 1 were smaller at second measurement occasion. This indicates that although on mean-level homogenization emerged, subgroups drifted into divergence between time-points. Overall, the level of interrater-agreement remained strong, but the tendency shown in the data is worth noting. It seems plausible that the interventions in the national improvement program only reached some individuals and not evenly addressed or reached all. One might suspect that without actively trying to interrupt this trend, groups might even drift further apart within locations. This, in turn, could lead to an overall weaker climate despite high mean levels in some subgroups. However, strong agreement has been proven to be an influential aspect of safety climate in order to prevent errors or adverse events. The shape of the ratings within groups suggests that no distinct subgroups emerged yet. However, subtle decreases in rwg-values indicate that formation of distinct subgroups might only be a matter of time unless specific interventions address cohesion of safety climate within groups.

The results for hospital 2 present a different picture concerning safety climate differences between time points. Overall, safety climate was significantly higher at second measurement occasion. Hence, across time-points and subgroups, safety climate ratings were higher, indicating better safety climate as perceived by hospital staff. Analysing subgroups, however, only safety climate in the OR but not on wards improved. This seems plausible as the intervention program mainly addressed staff working in the OR. Due to differences that only emerged in the OR, differences between ward and OR became significant at time-point two. Hence, concerning safety climate in different departments, divergence emerged at time point two. Although hospital two also had strong agreement ratings at both measurement occasions, tendencies in the data point to a stronger climate at second measurement occasion. Though departments drifted apart, within groups ratings became more homogeneous. Subgroups are more harmonized with respect to their climate ratings at the second measurement occasion. This also shows in the frequency histograms analysing shape. No distinct subgroups were evident within the rating-groups, confirming the notion of agreement within groups.

Taken results of both hospitals together in some subgroups significant differences in safety climate ratings emerged between time-points on mean-level. Also, the analyses of the additional dimension “strength” added valuable knowledge about differences across time. The analyses of strength further illustrate trends towards divergence or convergence within groups concerning perceptions of safety climate. This adds important information in order to understand differences within and between groups across time and may also point into fruitful directions for addressing climate issues within these hospitals. We believe that the present study illustrated the added value that is provided by the analyses of safety climate profiles instead of single measures. Taking several measures into account that describe safety climate from different perspectives is necessary in order to fully understand differences and trends within groups. The detailed picture that emerges from the analyses of different measures also allows for more specific interventions addressing the needs of different groups more precisely.

This study has several limitations. First, due to small samples, some differences could not be analysed (for example differences between ward and OR in hospital 1). This clearly represents a shortcoming of the present study as differences between ward and OR would have been expected. However, because the aim of the present study was to exemplify advantages of a full profile analyses instead of focussing only on mean-level, we believe that this limitation is acceptable. Second, significant differences that did emerge where all small. We can only speculate about the reasons, hence, clinical relevance of the differences found in the present study should be addressed in future studies. Third, data were not assessed longitudinally. It thus remains unknown whether differences between time-points reflect real changes in safety climate ratings or are rather due to sample differences. Though we cannot rule out this aspect completely, we believe that differences are likely to reflect true changes, as samples were comparable across time-points. Fourth, no inferences can be drawn from the present study concerning mechanisms underlying the changes in safety climate. Whether they were due to the intervention program or represent secular trends or even solely reflect individual preferences and characteristics and are, hence, random rather than systematic, remains speculative. Future studies need to address underlying mechanisms causing differences, in particular, changes in climate strength. A final shortcoming lies in the fact that results from the data are not generalizable across hospitals. However, analyses in the present study revealed important facts that need to be considered when analysing safety climate. We emphasize that analysing safety climate from different perspectives adds important additional knowledge to mean-level analyses. Also, taking different subgroups within a hospital into account is valuable. Although limited in generalizability the results of the present study thus add important knowledge to the literature on safety climate in hospitals.

## Supporting information

S1 Fig Frequency histograms of safety climate distribution. (DOCX)

S1 Table Items of the Safety Climate Survey. (DOCX)

S2 Table Original data of the survey. Presented are the raw data underlying the reported findings.
(XLS)
