# Supplementary figures and images for: Patient safety climate profiles across time: Strength and level of safety climate associated with a quality improvement program in Switzerland—A cross-sectional survey study
Source: PLoS One. 2017 Jul 28;12(7):e0181410. doi: 10.1371/journal.pone.0181410 (PMC5533316; doi:10.1371/journal.pone.0181410)

### S1 Figure: Frequency histograms of safety climate distribution

###
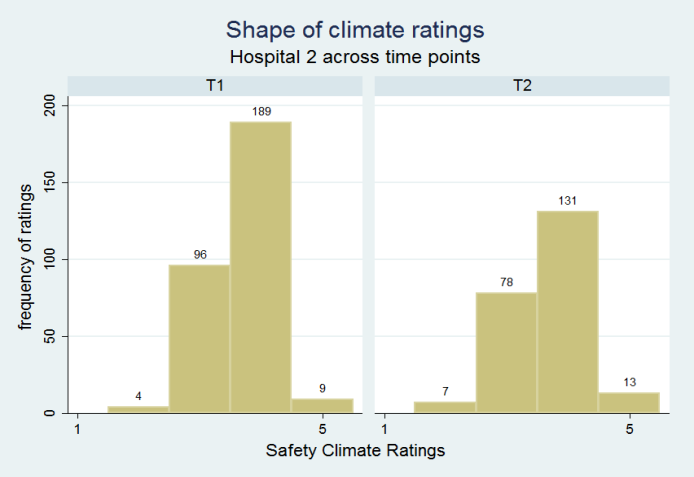

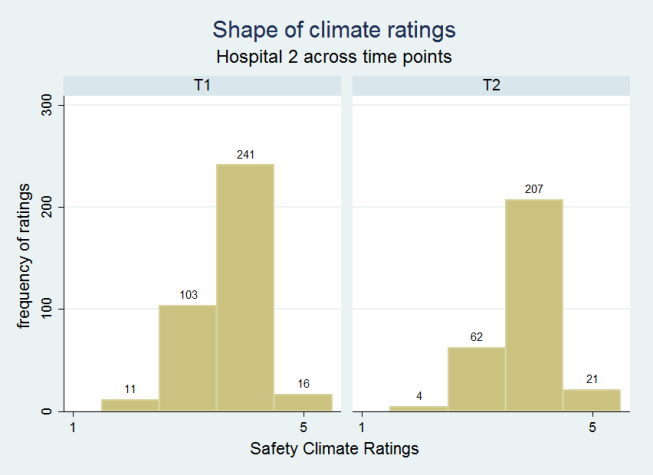

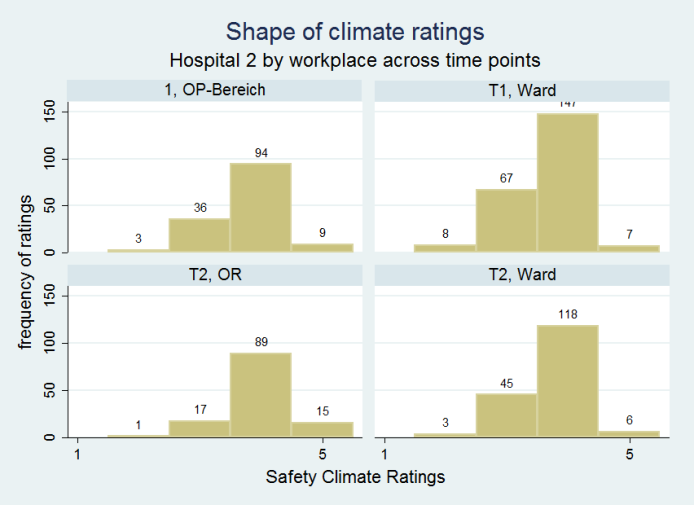

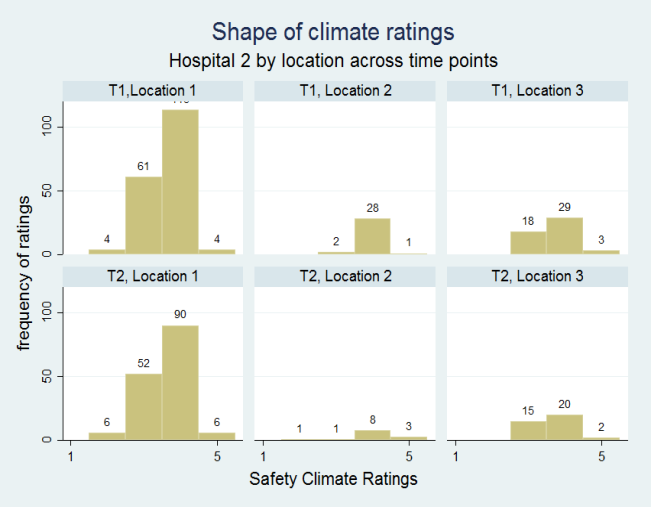


### T1 = Time-point 1, T2 = Time-point 2

Supplement: S1 Fig — (DOCX) [file pone.0181410.s001.docx]
