# Supplementary material for: Patient safety climate profiles across time: Strength and level of safety climate associated with a quality improvement program in Switzerland—A cross-sectional survey study
Source: PLoS One. 2017 Jul 28;12(7):e0181410. doi: 10.1371/journal.pone.0181410 (PMC5533316; doi:10.1371/journal.pone.0181410)
Supplement: S1 Table — (DOCX) [file pone.0181410.s002.docx]

**S1 Table: Items of the Safety Climate Survey**

| Item No. | Item |
| --- | --- |
| V1 | The culture of this clinical area makes it easy to learn from the mistakes of others. |
| V2 | Medical errors** are handled appropriately in this clinical area.  *** Medical error is defined as any mistake in the delivery of care, by any healthcare professional, regardless of outcome.* |
| V3 | The senior leaders in my hospital listen to me and care about my concerns. |
| V4 | The physician and nurse leaders in my area listen to me and care about my concerns. |
| V5 | Leadership is driving us to be a safety- centred institution. |
| V6 | My suggestions about safety would be acted upon if I expressed them to management. |
| V7 | Management/leadership does not knowingly compromise safety concerns for productivity. |
| V8 | I am encouraged by my colleagues to report any safety concerns I may have. |
| V9 | I know the proper channels to direct questions regarding patient safety. |
| V10 | I receive appropriate feedback about my performance. |
| V11 | I would feel safe being treated here as a patient. |
| V12 | Briefing personnel before the start of a shift (i.e., to plan for possible contingencies) is an important part of patient safety. |
| V13 | Briefings are common here. |
| V14 | I am satisfied with the availability of clinical leadership. |
| V14a | Physician |
| V14b | Nursing |
| V14c | Pharmacy |
| V15 | This institution is doing more for patient safety now than it did one year ago. |
| V16 | I believe that most adverse events occur as a result of multiple system failures and are not attributable to one individual’s actions. |
| V17 | The personnel in this clinical area take responsibility for patient safety. |
| V18 | Personnel frequently disregard rules or guidelines that are established for this clinical area. |
| V19 | Patient safety is constantly reinforced as the priority in this clinical area. |
